# Long COVID and NCDs: the Elephant in the Room

**DOI:** 10.1093/eurpub/ckac129.140

**Published:** 2022-10-25

**Authors:** I Nagyova

**Affiliations:** 1 Faculty of Medicine, Department of Social and Behavioural Medicine, PJ Safarik University, Kosice, Slovakia; 2 EUPHA

## Abstract

**Issue/problem:**

COVID-19 has now emerged as a public health concern that not only disrupted healthcare services of people living with non-communicable diseases (NCDs) but likely also increase the burden of NCDs.

**Description of the problem:**

Before the pandemic, SDG target 3.4, which calls for a reduction in premature mortality from NCDs by one third by 2030, was expected to be achieved by 17.5% of countries, and a further of 23% could achieve the target with a slight acceleration in decline. Currently, most countries are off track due to disruption of healthcare services for people with NCDs, overstretched health systems, and fewer resources than before the pandemic. In addition, only around one in four people who had COVID-19 reported feeling fully recovered within a year of being discharged from hospital. The adverse health effects from long COVID range from ‘invisible symptoms’ such as fatigue and difficulty concentrating, to neurological and neuropsychiatric symptoms, respiratory and cardiovascular problems, and metabolic disease.

**Results:**

The COVID-19 pandemic has shown that siloed programmes are increasingly unfit, and the bidirectional relationships between communicable diseases and NCDs underscore the need to dismantle disease-specific silos, emphasising reforms and investments that improve a wide range of health outcomes. Cost-effective, feasible, and relevant interventions that target both physical and mental health impairments are urgently required.

**Lessons:**

By 2030, ministries of health would need to contribute about 20% of their budgets to high-priority NCD interventions. Protecting current investments and scaling up cost-effective public health interventions is especially crucial in the context of long haul COVID and NCDs. Policy makers and planners will need concrete guidance for making progress on SDG target 3.4, often with overburdened health systems and scarcer resources.

